# Logic Gates Based on 3D Vertical Junctionless Gate-All-Around Transistors with Reliable Multilevel Contact Engineering

**DOI:** 10.1021/acs.nanolett.3c04180

**Published:** 2024-06-17

**Authors:** Abhishek Kumar, Jonas Müller, Sylvain Pelloquin, Aurélie Lecestre, Guilhem Larrieu

**Affiliations:** † LAAS-CNRS, Université de Toulouse, CNRS, 7 Avenue Colonel Roche, 31031 Toulouse, France

**Keywords:** nanoelectronics, vertical transport transistor, nanowire, scaled gate-all-around, logic gates, 3D device

## Abstract

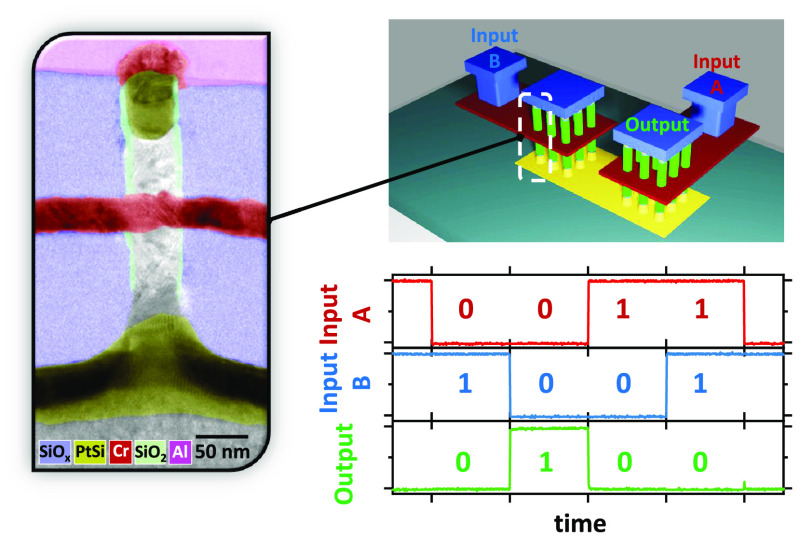

Vertical gate-all-around (V-GAA) represents the ultimate configuration in the forthcoming transistor industry, but it still encounters challenges in the semiconductor community. This paper introduces, for the first time, a dual-input logic gate circuit achieved using 3D vertical transistors with nanoscale sub-20-nm GAA, employing a novel technique for creating contacts and patterning metallic lines at the bottom level without the conventional lift-off process. This involves a two-step oxidation process: patterning the first field oxide to form bottom metal lines and then creating the gate oxide layer on nanowires (NWs), followed by selective removal from the top and bottom of the nanostructures. VGAA-NW transistors, fabricated using the lift-off-free approach, exhibit improved yield and reduced access resistance, leading to an enhanced drive current while maintaining good immunity against short-channel effects. Finally, elementary two-input logic gates within a single cell, using VNW transistors, demonstrate novel possibilities in advanced logic circuitry design and routing options in 3D.

The semiconductor industry is witnessing a significant transition toward planar vertically stacked gate-all-around (GAA) nanosheet transistors, marking the pinnacle of fin-field-effect-transistor (FinFET) evolution, particularly evident for the 2 nm technology node.^1,2^ Stacking multiple nanosheets offers enhanced current transport capabilities compared to similarly sized FinFETs.^3^ Moreover, GAA-based approaches effectively tackle short-channel effects.^4,5^ Notwithstanding their design flexibility, GAA nanosheets and GAA silicon nanowires (NWs) adhere to a planar layout for gate length (*L*_g_) and source/drain contact area, limiting overall circuit design possibilities.^6^ Nonetheless, these limitations can be mitigated through the vertical integration of gate-all-around nanowires (VGAA-NWs), offering several key advantages in processing and circuit design. Vertical gate length definition enables extension without altering the surface footprint, allowing for relaxed nanowire diameter while maintaining effective short-channel control.^7−10^ Scaling down the device enhances gate controllability, reduces power dissipation, and facilitates seamless integration into higher-density arrays.^11,12^ To advance vertical designs using nanowires, achieving low-resistance contacts on both ends independently is crucial. This is essential for realizing future nanowire-based digital circuitry devices, enabling fabrication of intricate structures integrating multiple devices into logic cells, which is essential for data processing, arithmetic operations, and information storage. Although VGAA-NWs show great potential for high-performance devices as has been highlighted by simulation^13−15^ and experimental demonstrations,^16−18^ the majority of device demonstrations lack symmetrical bottom and top contacts, limiting integration capabilities. The challenge lies in contact fabrication, where conventional methods (such as lift-off patterning) suffer from degraded contact quality, especially in multilayer processes, where the top and bottom contacts of vertical nanowires are fabricated in sequence. Hence, previous VGAA-FET fabrications have omitted bottom contacts on the sample substrate to simplify the process.^7,11,12,19,20^ A novel approach is needed for source and drain contact fabrication to avoid asymmetrical contacts negatively impacting device performance. In this Letter, we present a large-scale microelectronics process for manufacturing GAA vertical silicon nanowire MOSFETs, introducing a novel lift-off-free method for creating symmetrical top and bottom contacts for nanowires. This method significantly reduces contact resistance and enhances the device yield. Employing an etchback technique^21,22^ for structuring all metal layers in the entire fabrication process, we demonstrate optimized fabrication of p-type VNW-FETs with sub-20-nm gate length. We provide a detailed discussion of their electrical performance, showcasing an important milestone in fully vertical technology: the integration of elementary two-input logic gates (NOR and NAND) within a single cell, underscoring the robustness of this technology.

In contrast to prior research studies,^8,10^ we propose a novel approach for the concurrent production of bottom and source contacts on vertical nanowires without metal patterning by a lift-off approach. We utilize a two-step oxidation process, first patterning the first field oxide to create the bottom metal lines, followed by local oxide removal to establish the gate oxide layer on the nanowires, and then selectively removing it from the top and bottom of the nanowires. Instead of using the conventional lift-off process for metal deposition, we adopt the etchback technique commonly used in industrial semiconductor fabrication to deposit a full metal layer on the entire sample. This ensures perfect contact between the conductor and the nanowires. The sample is annealed to form metal–silicide contacts only on the oxide-free top and bottom contacts, preventing the reaction of the metal layer with the oxide. The remaining unreacted metal is selectively removed through chemical wet etching, leaving silicided contacts behind. The technique enables the creation of nanometer-scale contacts using industrial μm-patterning techniques and can be applied to other metal layer depositions, such as the gate layer or additional contacts, using conventional lithographic masks. The remaining undesired metal layer can be stripped without leaving any traces or defects. Even if the presented demonstration is performed on a silicon nanowire channel, the process route can be directly applicable to other semiconducting materials as such high-mobility SiGe nanowires^23^ and III–V materials, like GaAs and GaSb.^24,25^

The complete fabrication process of such a vertical FET device is illustrated in Figure 1. Initially, vertical silicon NWs are formed using a top-down approach, where circular nanopillars are patterned in negative-tone resist (hydrogen silesquioxane, HSQ) through electron beam lithography. However, their final dimensions are obtained in two steps. First, the patterns are transferred through anisotropic reactive ion etching (Figure 1-I) onto the p-type Si substrate, achieving a desired nanowire height of around 200 nm. The diameters of the nanopillars are designed to be large enough to obtain final nanowire dimensions after a sacrificial wet oxidation step performed at 850 °C (Figure 1-II), simultaneously removing etching-induced defects. Therefore, a lithography step defines the bottom contact area containing the nanowire arrays and protects the rest of the substrate. Subsequently, when the wafer is chemically etched with buffered oxide etchant (BOE), the nanowires are also shrunk down to smaller diameters (down to 16 nm) and the substrate on the “bottom contact” is revealed. Next, a 4-nm-thick gate oxide (SiO_2_) is grown by thermal dry oxidation at 725 °C, followed by a precisely controlled anisotropic reactive ion etching (RIE) step that removes the oxide layer at the top and bottom of the NWs without altering the gate oxide on the sidewalls (Figure 1-III). To create the top and the bottom contacts of the NWs, a 10-nm-thick platinum (Pt) layer is deposited and activated through rapid thermal annealing (RTA) at 500 °C for 3 min (Figure 1-IV). The silicidation reaction occurs only where Pt is in direct contact with clean Si, resulting in platinum silicide (PtSi) on the source (bottom) and drain (tips of NWs). Any unreacted Pt on oxidized surfaces (including NW walls) is then simply removed by chemical etching in aqua regia. A dielectric spacer is formed (Figure 1-V) using HSQ as a spin-on-glass (SOG) with a low dielectric constant (*k* < 3) where the thickness is adjusted by chemical etching in diluted hydrofluoric acid.^26,27^ The metallic gate is deposited on the whole surface by evaporation, and the device gate length is finely adjusted by the deposited metal thickness (Figure 1-VI). An 18-nm-thick Cr layer is deposited to form the gate-all-around in the middle section of the Si NW channels. The layout of the layer is defined by conventional lithography and a selective Cr etchback process. A second HSQ spacer is deposited on top of the devices to completely cover the NWs (Figure 1-VII). The spin-on-glass is thermally cured under forming gas (FGA) and etched down to the height of the silicide contacts on top of the NWs. To access the bottom PtSi and the Cr gate layer, vias are defined by lithography and plasma-etched through the spacers. Finally, a 400-nm-thick aluminum layer is deposited, filling the via holes, and patterned afterward to form the contact pads by wet Al etchback (Figure 1-VIII). Finally, the sample is annealed in an RTA process under a forming gas atmosphere of N_2_/H_2_ (96%/4%) at 200 °C for 4 min to passivate defects at both the Si–SiO_2_ interface and at the Si–PtSi contact interface of the NWs.

**Figure 1:**
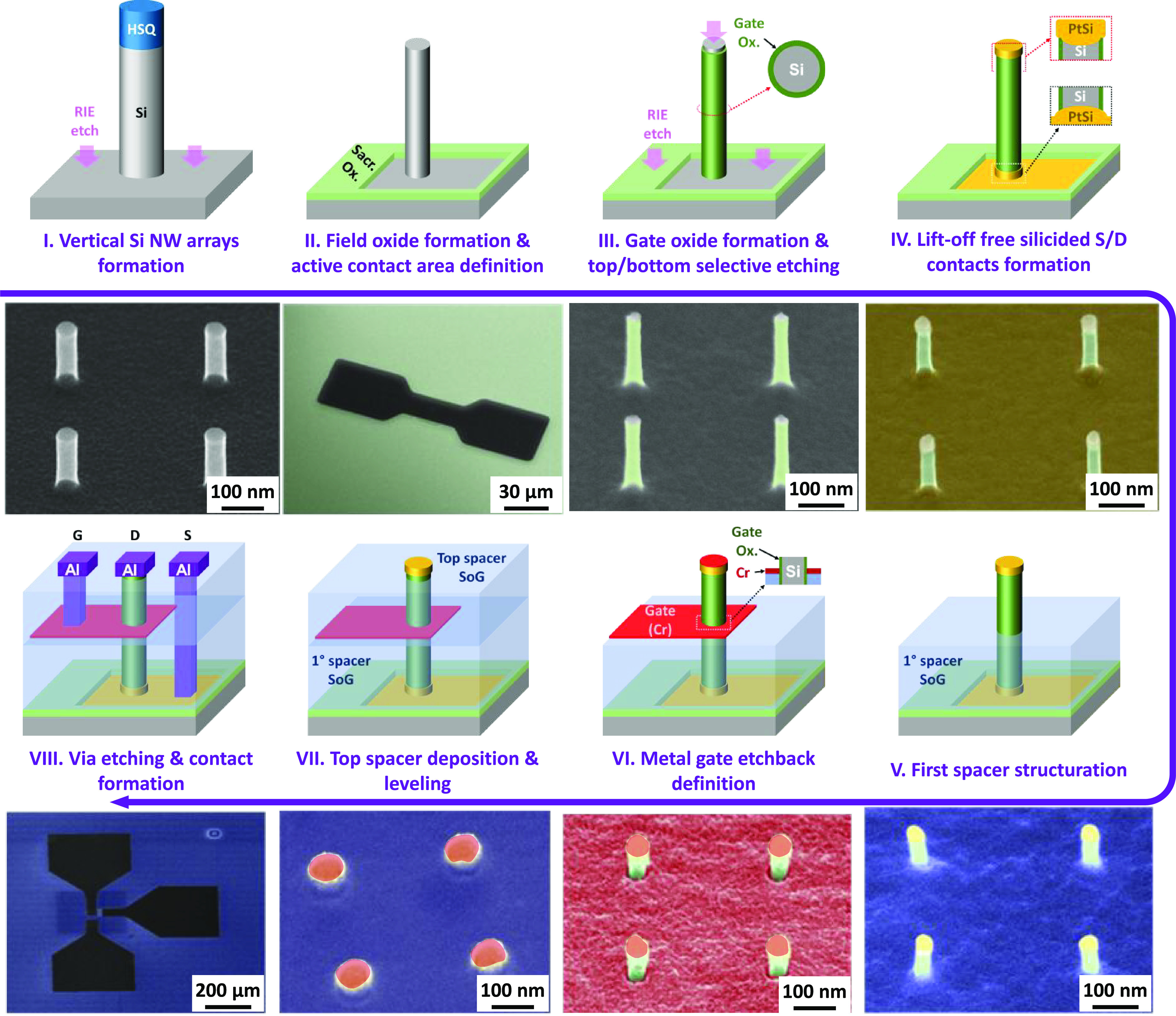
Schematic 3D illustration and tilted SEM image for every step of the fabrication flow for VGAAFET using etchback contact patterning without classical lift-off processes, which involves depositing a metal layer through an organic resist matrix to obtain a metallic pattern, frequently used in small-scale academic fabrications: I. Vertical Si NW array obtained by top-down fabrication. SEM image displays cleaned nanowires. II. Wet oxidation, lithography, and sacrificial oxide etching with shrinkage of the NWs. III. Gate oxide formation and S/D etchback. IV. Silicided source/drain contact formation. V. First dielectric (SiOx) spacer formation. VI. Metal gate deposition and etchback structuration using a commercial Cr wet-etchant solution (TechniEtch Cr01 from MicroChemicals). VII. Top dielectric (SiOx) spacer formation. VIII. Milling and deposition of an aluminum via and extrinsic contact pads (S/D/G), followed by annealing.

After fabrication, a transmission electron microscopy analysis (Figure 2b) reveals the device’s 3D composition (Figure 2a). The Cr-GAA layer encircles the NW, forming a circular shape due to the observation angle. A distinct spacing corresponding to the gate oxide layer is visible along with a homogeneous HSQ layer thickness, devoid of defects or wave effects. Characterization of a single channel using EDX and STEM microscopy (Figure 2 c and d) illustrates the Si nanowire (cyan) surrounded by the oxygen-rich SiO_*x*_ spacer layer (green) and chromium GAA (red), with aluminum top contact (magenta). Detailed STEM imaging highlights the three conductive layers of the VNW-FET (source, gate, and drain contacts), showing Pt deposition penetrating the NW to form PtSi, with a deeper penetration at the bottom. Asymmetrical spacer layers are crucial to center the GAA within the Si channels.

**Figure 2:**
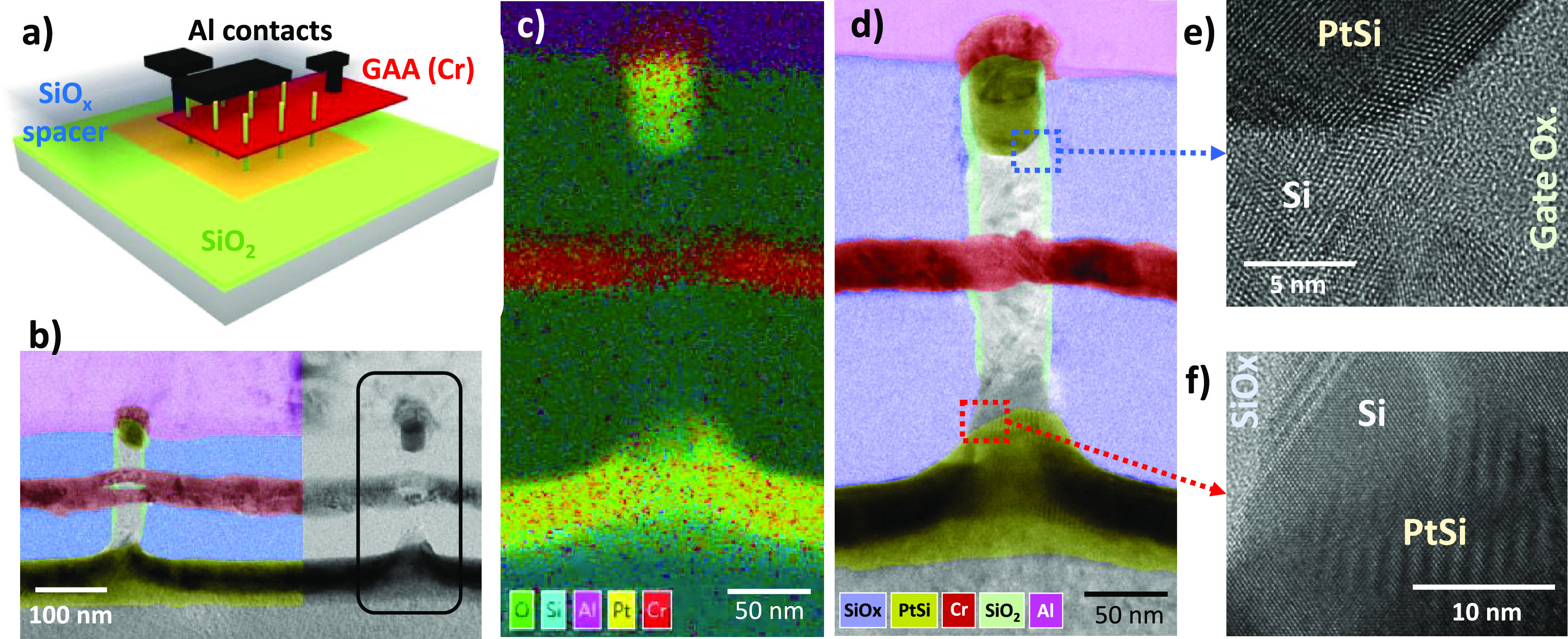
(a) Schematic aerial view of a vertical FET device implemented on a dense nanowire array with extrinsic access connecting different levels. (b) TEM cross-section of the nanowire array in tilted view. A single nanowire, framed in (b), has been analyzed by EDX (c), and the magnified TEM image (d) has been colorized with false colors to show the different layers of the device: the gate surrounding each nanowire, symmetrical silicided S/D (PtSi) contacts, and 80-nm-thick low-*k* dielectric spacers separating the S/D contacts from the gate. Detailed views of source and drain regions are respectively shown in (e) and (f). The EDX map in (c) has been recorded over a short acquisition period to limit image drift, thus exhibiting artificial Cr signals in the PtSi layers.

Electrical characterizations at room temperature of such vertical NW architectures with a nominal gate length of 18 nm, 210 nm high NWs, and diameters between 16 and 34 nm are shown in Figure 3 with static characteristics (*I*_D_–*V*_G_ and *I*_D_–*V*_D_). The devices exhibit high drive currents (at *V*_DS_ = −1.1 V, |*V*_G_ – *V*_t_| = 1 V, normalized by the number and the diameter of NWs) of 70, 477, and 665 μA/μm when the NW diameters are 16, 27, and 34 nm, respectively. With the increasing NW diameter, the strength of the gate control on the channel is decreasing, as described by the *I*_on_/*I*_off_ ratio: while 16 and 27 nm NW-diameter transistors show good gate control with ratios on the order of 10^6^ (at *V*_DS_ = −0.1 V), the electrostatic control of the gate degrades quickly for NW diameters larger than 30 nm, and the device cannot be turned off at zero gate bias. Another observable change for smaller NWs is the shift of the threshold voltage (*V*_th_) at the onset of the linear regime of the *I*_D_–*V*_G_ plots. In junctionless nanotransistors, the threshold voltage is determined by the extent of the depletion region, rather than by the formation of accumulation or inversion layers.^28^ The threshold voltage can be regarded as the gate bias level at which bulk conduction initiates. This criterion is met when there exists a specific point within the channel’s center that remains undepleted,^29^ and this point varies depending on the device’s nanowire diameter.

**Figure 3:**
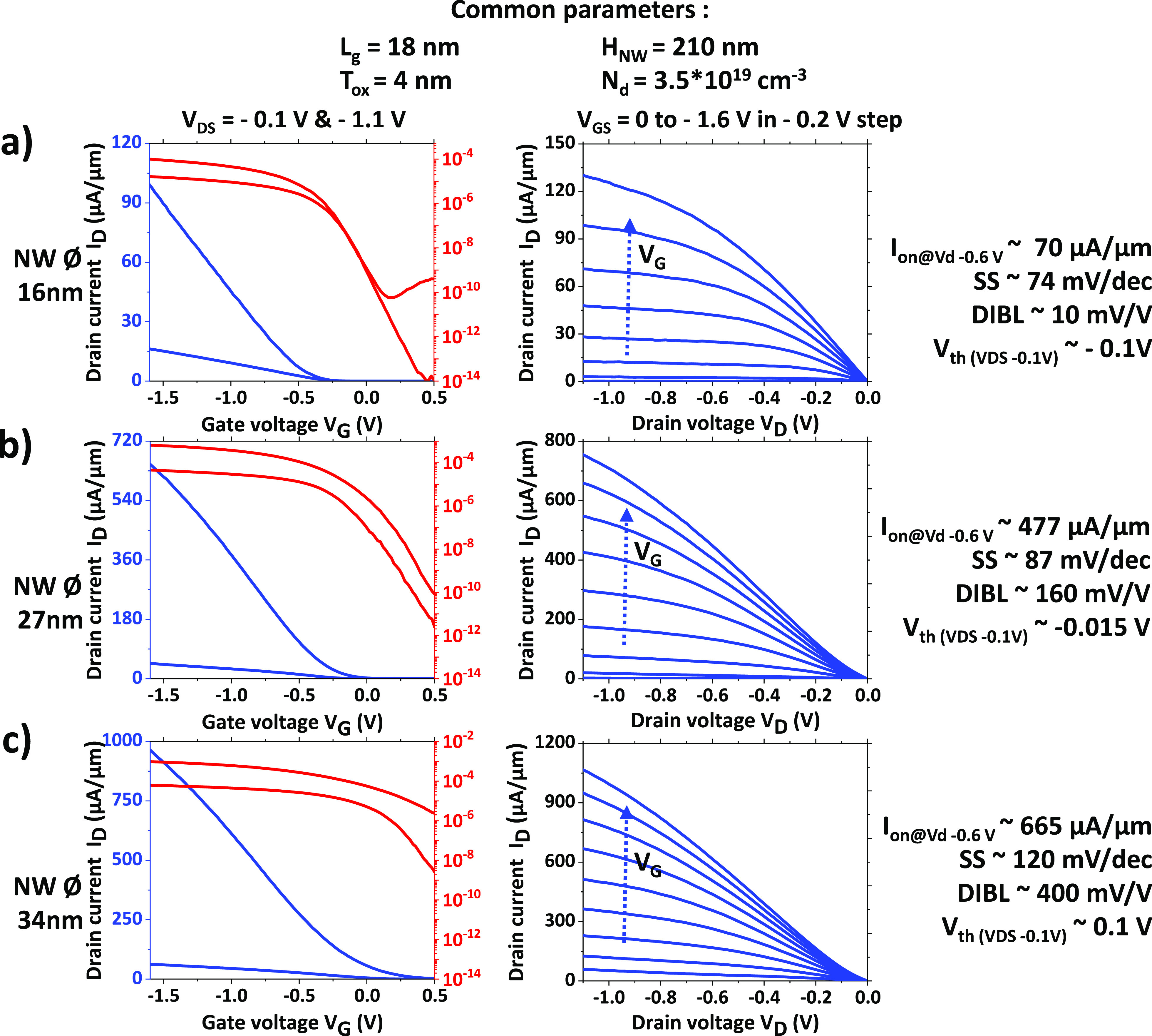
Transfer and output characteristics of a junctionless GAA p-type MOSFET on a Si VNW with a diameter of (a) 16 nm, (b) 27 nm, and (c) 34 nm performed with a Cascade prober and parametric analyzer “Agilent 4156C”. The drain current over the gate voltage (*I*_D_–*V*_G_) is plotted in both linear (blue) and logarithmic scale (red) for different drain voltages, as well as the drain current over the drain voltage (*I*_D_–*V*_D_) for varying gate bias.

To contextualize our device characteristics, we reference the projected properties for n-type lateral GAA 3D devices in the International Roadmap for Devices and Systems.^30^ Our device, featuring a 27 nm NW diameter and an 18 nm gate length, achieves an *I*_D_ of 390 μA/μm with *I*_off_ = 11 pA/μm and *V*_D_ = −0.6 V, aligning closely with projections for lateral GAA 3D devices with a 12 nm gate length in the 2031 generation, which anticipate an *I*_D_ of 459 μA/μm at *I*_off_ = 100 pA/μm and *V*_D_ = 0.6 V. Despite p-type devices typically underperforming n-type counterparts, our results demonstrate comparable performance.

The proposed GAA devices are expected to exhibit strong immunity to short channel effects (SCEs) thanks to their gate-all-around configuration, effectively depleting the doped channel at zero bias. The quality of gate control under applied gate bias can be assessed through the subthreshold slope (SS) and drain-induced barrier lowering (DIBL), as depicted in Figure 4a. DIBL is defined as the change in threshold voltage (*V*_G_) when the drain voltage increases from −0.1 V to −1.1 V, while the SS represents the slope in the linear regime on a logarithmic scale. For instance, our device with a 16 nm NW diameter demonstrates a normally off state at zero bias, featuring nearly ideal subthreshold characteristics (SS ∼ 74 mV/dec and DIBL ∼ 10 mV/V). As the NW diameter increases, both SS and DIBL values escalate due to electrostatic control degradation during normally off operation. However, we observe that SS values can be maintained close to the theoretical limit (SS_th_ ∼ 60 mV/dec) for VNW-GAA devices with highly doped substrates when the nanowire diameter is 27 nm or less.

**Figure 4:**
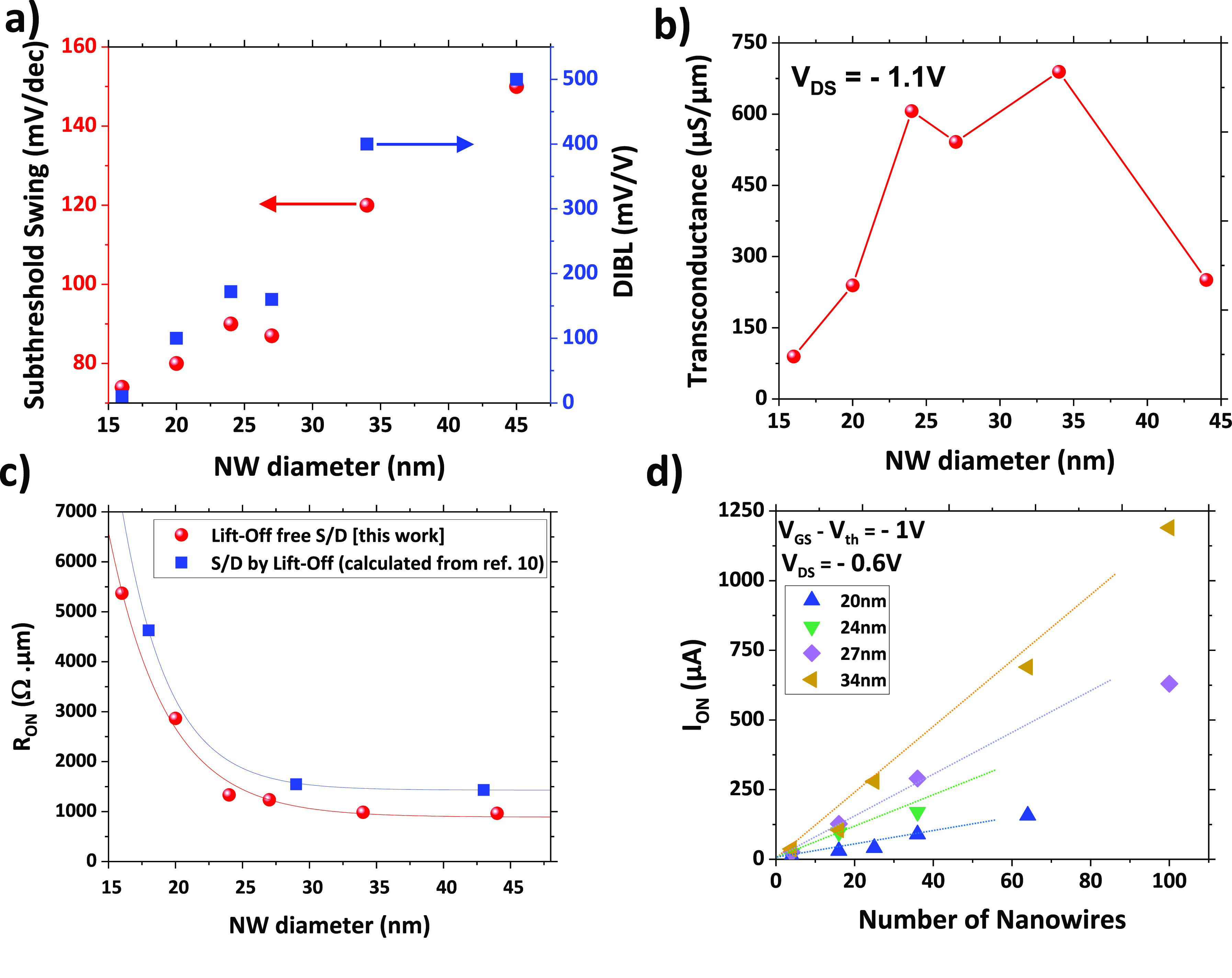
Subthreshold swing (SS) and DIBL (a) and transconductance *G*_m_ (b) as a function of the NW diameter. (c) Comparison of ON resistance (*R*_ON_) as a function of the NW diameter for the lift-off-free source/drain approach (this work) versus the lift-off technique used in previous work (calculated from ref (10)). Scalability of the VT-FETs was represented as *I*_ON_ as a function of the number of NWs used (d).

Furthermore, the increase in the drain current with *V*_GS_ and the device amplification amount can be characterized through transconductance (*G*_m_). In a FET, it is defined by the following relation:
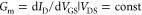
1

Figure 4b presents the transconductance for various NW diameters using eq 1, revealing a nonlinear trend. Initially, the transconductance rises as the NW diameter increases, primarily due to the heightened drive current associated with larger NW diameters. This increase in the drive current is attributed to the reduced access resistance resulting from a highly doped channel. However, as the diameters exceed 34 nm, electrostatic control diminishes, consequently reducing the device’s *I*_on_/*I*_off_ ratio. Consequently, we observe a notable change in the transconductance slope, reaching its peak at an NW diameter of approximately 34 nm before declining beyond this point.

To evaluate the improvements resulting from our lift-off-free etchback process, we conducted an ON resistance extraction for various diameters, as presented in Figure 4c. These measurements were then compared to data obtained from similar structures created using traditional lift-off methods^11^ (indicated as blue squares). The findings clearly demonstrate that the etchback technique significantly reduces device resistance (*R*_ON_ is estimated at 500 ± 40 Ω/μm for diameters ranging from 20 to 45 nm), emphasizing the pivotal role of contact quality. The substantial reduction in *R*_ON_ (for example, 26% of reduction for 27 nm NW diameter) can be attributed to the decrease in access resistance achieved through the new lift-off-free approach, as the channel configuration (and the associated channel resistance) remains similar in both architectures.

In our fabrication process, both the electrical performance and the available footprint are notably influenced by the quantity and diameter of the nanowires employed. We measured the drive current (*I*_ON_) at *V*_DS_ = −0.6 V (*V*_GS_ – *V*_Th_ = −1 V) across nanowires spanning diameters from 17 to 34 nm, as illustrated in Figure 4d. As anticipated, the drive current increases with larger nanowire diameters and with a greater number of nanowires at a consistent diameter. Intriguingly, a robust linear correlation between the drive current and the number of nanowires is observed, highlighting the ease with which electrical characteristics can be tailored by adjusting the number of nanowires. This relationship demonstrates the reliability and reproducibility of our devices as each nanowire in an array behaves like an individual transistor connected in parallel to the others. Furthermore, this indicates that our lift-off-free process yields uniformly accessible contacts.

Demonstrating the operational principles of logic gates with advanced devices is of paramount importance, as it showcases the practical application of cutting-edge technology in digital circuits. These demonstrations validate the feasibility of integrating these advanced devices into real-world computing systems, paving the way for more efficient and powerful electronics. p-Type VNW-FETs are integrated into a “pull-up network” (PUN) that links the logic gate output to the positive supply voltage. Additionally, a resistor is placed between the logic gate output and the negative supply voltage. This configuration ensures that when the desired output is in a high state, the PUN activates, allowing the current to flow from the positive supply to the output. Figure 5 illustrates the first implementation of a passive NOR logic gate constructed on a p-type substrate. This system comprises two VNW-FETs connected in series, with each of them composed of a 25-NW array and with each nanowire having a diameter of 24 nm. The gates, featuring 18-nm-length Cr, are linked to inputs A and B, and top contacts are employed to adjust the load resistance and monitor the output, as outlined in the electrical diagram and depicted in the 3D representation. Pulses of 0.5 s are alternately applied to inputs A and B using a Tektronix AFG3102 to replicate the truth table of a p-NOR gate (as shown in Figure 5f). It is important to note that the system operates in a negative logic configuration, where logic “0” corresponds to −1 V and logic “1” to 0 V. The voltage *V*_D_ is provided through a load resistance *R*_L_ and measured with an Agilent DSOX3034A oscilloscope, featuring an *R*_o_ of 1 MΩ impedance. *V*_D_ is then adjusted to achieve a minimum *V*_Y_ of −1 V . The results depicted in Figure 5d and e were acquired by using a load resistance of 1 MΩ. The median values for *V*_Y_ being “0” and “1” are measured at approximately −1.022 and −0.040 V, respectively, resulting in an almost ideal logic response. of 98.2%.

**Figure 5:**
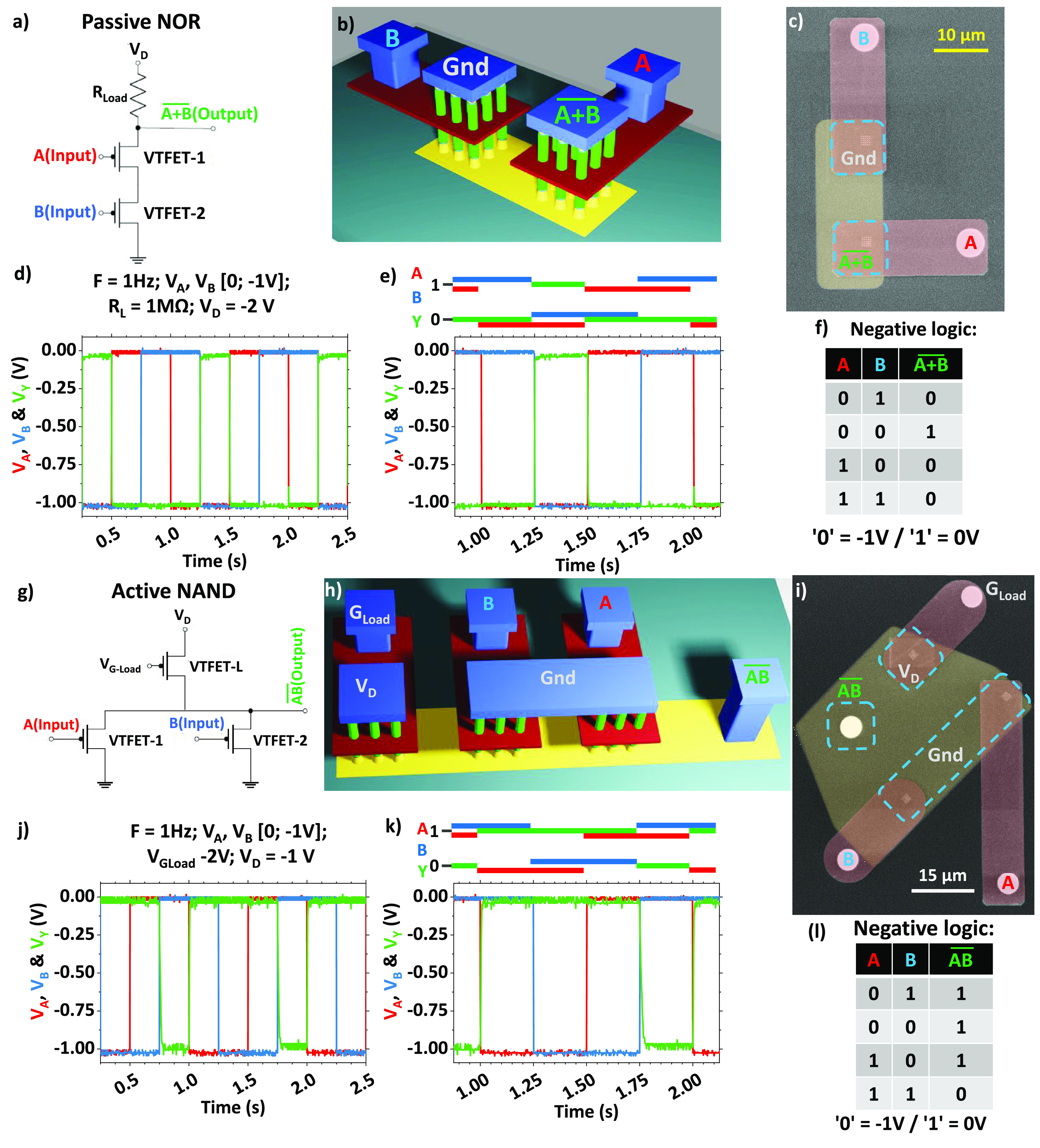
Electrical diagram (a), 3D representation (b), and SEM top view before contact formation (c) of a passive NOR fabricated with two VNW-FETs connected by the bottom contacts. A/B inputs and  output are shown in (d) for 3 cycles and emphasized in (e), and the truth table is visible in (f). Electrical diagram (g), 3D representation (h), and SEM top view before contact formation (i) of an active NAND fabricated with three VNW-FETs connected by the bottom contacts. A/B inputs and  output are shown in (j) and emphasized in (k), and the truth table is visible in (l).

A second demonstration involved even more complex systems, testing a functional active NAND gate, as presented in Figure 5g, h, and i. This device comprises three VNW-FETs with a common bottom contact, responding to the truth table depicted in Figure 5l (lower right). The V-FETs are 5 × 5 arrays of 24-nm-diameter NWs in a p-doped Si substrate. A/B inputs are polarized with alternating 0.5 s and −1 V pulses, such that an “ON” state can only be detected in the output when both A and B are in the “OFF” state. The gate voltage of the load transistor (*V*_G-load_ = −2 V) was adjusted to achieve the optimal signal, where the  is 94.5%, as shown in Figure 5j.

This demonstration reveals the feasibility of dual-input logic gates using 3D vertical nanowire transistors, opening up new possibilities for advanced logic circuit design. In particular, signal routing within the device capitalizes on the diverse metallic planes distributed vertically, offering versatile routing options. This leads to shorter connection tracks between individual transistors and enables the creation of denser, high-performance logic devices.^31^ An initial indication of the benefit in terms of surface area can be obtained by comparing the vertical 3D integration of the passive NOR gate presented in Figure 5 with the equivalent small circuit realized using planar technology, as shown in the Supporting Information in Figure S1. In comparison, vertical integration leads to an approximate footprint reduction of more than 50%. This is in line with other studies,^32^ which show that compared to planar 7 nm FinFET technology, VNWFET-based complementary inverters achieve a 48% reduction in lateral dimensions and an even more substantial 84% reduction when considering only the active part of the layout.

In conclusion, this letter introduces a dual-input logic gate circuit (passive NOR and active NAND) achieved using 3D vertical transistors with nanoscale GAA. To achieve this, we propose a large-scale process for manufacturing Si VNW MOSFETs with an 18-nm-thick nanoscale GAA, based on a novel technique for creating contacts and patterning metallic lines at the bottom level without the conventional lift-off process. It involves a two-step oxidation process: first, patterning the first field oxide to form bottom metal lines and then locally removing the oxide to establish the gate oxide layer on nanowires, followed by selective removal from the top and bottom of the nanowires. VGAA-NW-FETs, fabricated using the lift-off-free approach, exhibit an improved yield and reduced access resistance. We observed an enhanced drive current for larger NW diameters and excellent immunity against short channel effects in nanowires with diameters below 34 nm, making them suitable for high-density applications as compared to the international roadmap predictions. While larger NWs experience a degradation of electrostatic control and *I*_on_/*I*_off_, their substantial increase in drive current makes them interesting for high-power applications. This breakthrough in constructing dual-input logic gates with 3D vertical transistors opens the door to advanced logic circuitry design, leveraging the full potential of the device’s 3D configuration.

## Supporting Information Available

Planar vs vertical configuration: cell area comparison of NOR logic gates (PDF)
