# Supplementary material for: Logic Gates Based on 3D Vertical Junctionless Gate-All-Around Transistors with Reliable Multilevel Contact Engineering
Source: Nano Lett. 2024 Jun 17;24(26):7825–32. doi: 10.1021/acs.nanolett.3c04180 (PMC11229076; doi:10.1021/acs.nanolett.3c04180)
Supplement: Supplementary file 1 — nl3c04180_si_001.pdf [file nl3c04180_si_001.pdf]

Supporting Information

Logic Gates Based on 3D Vertical Junction-Less Gate-All-Around Transistors with Reliable Multi-Level Contact Engineering

Abhishek Kumar [a], Jonas Müller [a], Sylvain Pelloquin [a], Aurélie Lecestre [a], Guilhem Larrieu [a,*]

[a] *LAAS-CNRS, Université de Toulouse, CNRS,* 7 av. Colonel Roche, 31031 Toulouse, France.

**E-mail : guilhem.larrieu@laas.fr*

Planar vs. Vertical Configuration: Cell Area Comparison of NOR Logic Gates

The vertical 3D integration of a passive NOR gate using the JL-VNWFET architecture is compared to its corresponding 2D projection realized in planar technology with the same gate length and width, as shown in Figure S1, to estimate the relative reduction in the logic gate's cell area (F^2). The presented layouts follow the classical design rules [1, 2], with F defined as the minimal lithographic feature of the densest process layer. The minimum distance between two contacts is defined as the same as the minimum contact size (F), and the minimum space between the gate and contact is fixed at 2/3 of F.

This comparison yields an approximate cell size reduction of 53%, due to the more compact in-line arrangement of the S/D and gate contacts in the vertical layout. Furthermore, the vertical integration provides additional advantages: (i) the gate length (L_G) is defined vertically, allowing a relaxation of the dimension scaling without surface penalty; (ii) insulation wells (green areas) can easily be arranged under gate contacts without using additional surface area; (iii) vertical integration is more favorable for minimizing unused surface area (white surface).

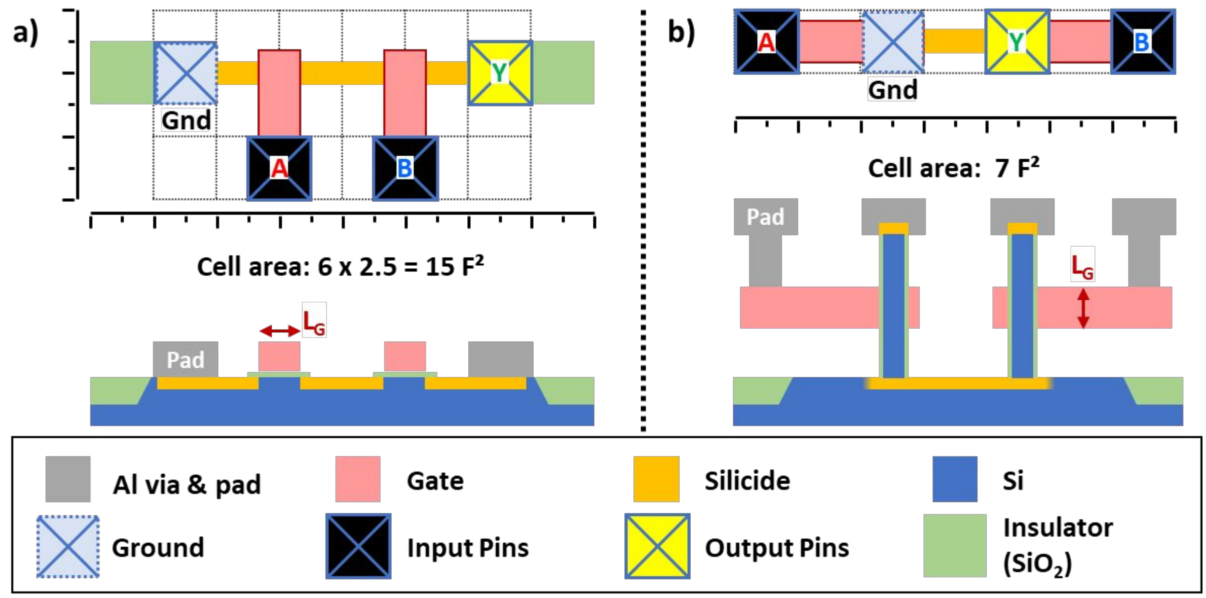

Figure S1: Schematic views of the same passive NOR cell on p-MOS-only logic compared between (a) a classical planar architecture and (b) our Vertical Transport Junction-Less architecture. Cell areas are expressed in F^2 units; F being the minimal lithographic feature of the densest process layer.

References

[1] T. Cui, Q. Xie, Y. Wang, S. Nazarian and M. Pedram "7nm FinFET Standard Cell Layout Characterization and Power density Prediction in Near- and Super-Threshold Voltage Regimes", Int. Green Computing Conf. (IGCC), 2014. doi: 10.1109/IGCC.2014.7039170

[2] C. Mukherjee, M. Deng, F. Marc, C. Maneux, A. Poittevin, I. O'Connor, et al. "3D Logic Cells Design and Results Based on Vertical NWFET Technology Including Tied Compact Model", 2020 IFIP/IEEE 28th International Conference on Very Large Scale Integration (VLSI-SOC). doi: 10.1109/VLSI-SOC46417.2020.9344094
